# Thrombocytopenia in the intensive care unit: diagnosis and management

**DOI:** 10.1186/s13613-025-01447-x

**Published:** 2025-02-22

**Authors:** Frédéric Pène, Lene Russell, Cécile Aubron

**Affiliations:** 1 Service de Médecine Intensive – Réanimation, Hôpital Cochin, Assistance Publique-Hôpitaux de Paris. Centre, Université Paris Cité, 27 rue du Faubourg Saint-Jacques, 75014 Paris, France; 2 Institut Cochin, INSERM U1016, CNRS UMR8104, Université Paris Cité, Paris, France; 3 Department of Intensive Care, Copenhagen University Hospital Gentofte, Hellerup, Denmark; 4 Department of Clinical Medicine, University of Copenhagen, Copenhagen, Denmark; 5 Service de Médecine Intensive – Réanimation, CHU de Brest, Université de Bretagne Occidentale, Brest, France

## Abstract

**Background:**

This narrative review aims to describe the epidemiology and aetiologies of thrombocytopenia in critically ill patients, the bleeding risk assessment in thrombocytopenic patients, and provide an update on platelet transfusion indications.

**Results:**

Thrombocytopenia is a common disorder in critically ill patients. The classic definition relies on an absolute platelet count below 150 × 10^9^/L. Alternatively, the definition has extended to a relative decrease in platelet count (typically within a range of >30–>50% decrease) from baseline, yet remaining above 150 × 10^9^/L. Thrombocytopenia may result from multiple mechanisms depending upon the underlying conditions and the current clinical setting. Regardless of the causes, thrombocytopenia accounts as an independent determinant of poor outcomes in critically ill patients, albeit often of unclear interpretation. Nevertheless, it is well established that thrombocytopenia is associated with an increased incidence of bleeding complications. However, alternative factors also contribute to the risk of bleeding, making it difficult to establish definite links between nadir platelet counts at the expense of potential adverse events. Platelet transfusion represents the primary supportive treatment of thrombocytopenia to prevent or treat bleeding. As randomised controlled trials comparing different platelet count thresholds for prophylactic platelet transfusion in the ICU are lacking, the prophylactic transfusion strategy is largely derived from studies performed in stable haematology patients. Similarly, the platelet count transfusion threshold to secure invasive procedures remains based on a low level of evidence. Indications of platelet transfusions for the treatment of severe bleeding in thrombocytopenic patients remain largely empirical, with platelet count thresholds ranging from 50 to 100 × 10^9^/L. In addition, early and aggressive platelet transfusion is part of massive transfusion protocols in the setting of severe trauma-related haemorrhage.

**Conclusion:**

Thrombocytopenia in critically ill patients is very frequent with various etiologies, and is associated with worsened prognosis, with or without bleeding complications. Interventional trials focused on critically ill patients are eagerly needed to better delineate the benefits and harms of platelet transfusions.

## Introduction

Platelets are non-nucleated cells derived from megakaryocytes, the giant precursor cells within the bone marrow. They have a lifespan of 7–10 days, and their count is maintained at a steady state balanced by bone marrow production. It is noteworthy that platelets undergo circadian quantitative and functional variations in healthy subjects. During the day, platelet counts increase by 5%, and activation patterns are more pronounced in the morning [1].

Platelets are mainly recognised for their haemostatic role, which relies on the number of circulating platelets and their functional integrity. They patrol the vessel walls and become adherent to altered surfaces when activated by von Willebrand factor (vWF) and collagen. Once activated, platelets release their granules’ contents, including adenosine diphosphate (ADP), to recruit additional circulating platelets and amplify platelet activation and aggregation, thereby contributing to the haemostatic clot. Furthermore, platelets are immunomodulatory cells, owing to their critical roles in the rolling and diapedesis of neutrophils towards inflammatory sites and by deploying neutrophil extracellular traps to contain local infectious processes. Additionally, platelet activation is part of various pathogenic thrombotic mechanisms, including the immunothrombosis seen in the context of a dysregulated immune response [2, 3].

Thrombocytopenia is common in critically ill patients, though its significance is complex and depends on prior comorbid conditions and ongoing pathogenic processes. It is also associated with worse outcomes, including an increased risk of bleeding. In addition to treating the underlying disorder, managing thrombocytopenia often involves supportive measures, primarily platelet transfusion.

This narrative review aims to describe the epidemiology and aetiologies of thrombocytopenia in critically ill patients, discuss the risk of bleeding in thrombocytopenic patients, and provide an update on indications for platelet transfusion.

## Epidemiology of thrombocytopenia in critically ill patients

Over 70 studies have been published over the past 40 years reporting on the frequency of thrombocytopenia in the ICU. In most studies, the diagnosis of thrombocytopenia typically relies on an absolute platelet count below 150 × 10^9^/L, which corresponds to the classification in the SOFA score system as mild, moderate, severe and very severe when below 150, 100, 50 and 20 × 10^9^/L, respectively [4]. Platelet count is a dynamic variable in critically ill patients and typically decreases over the first days to reach a nadir around days 4–5 following ICU admission [5, 6]. Therefore, the definition of ICU-related thrombocytopenia has been broadened to also include a relative decrease in platelet count (typically within a range of >30–>50% decrease, yet remaining above 150 × 10^9^/L) [7]. The prevalence in reported studies ranges from 12 to 77%, and the overall incidence from 8 to 56 per 100 ICU admissions [7]. This variation in previous studies is largely explained by most being small, retrospective and single-centre, and because definitions of thrombocytopenia varied considerably. Recently, the large prospective international PLOT-ICU cohort study has provided a global view of thrombocytopenia in the ICU [8]. The study included 1168 patients from 52 ICUs in 10 countries, mainly medical patients with a relatively high number of haemato-oncological diseases (14.4%), and excluded patients admitted to the ICU for post-operative monitoring after elective surgery. The frequency of any thrombocytopenia was 43.2% (95% CI 40.4–46.1), distributed into baseline and ICU-acquired thrombocytopenia in 23.4% (20.0–26.0) and 19.8% (17.6–22.2), respectively [8].

Observational studies have reported high variability in the risk factors for developing thrombocytopenia in the ICU; however, comparing risk factors across studies is challenging as case mix, definitions of thrombocytopenia and analytic methods differ. Nevertheless, higher disease severity scores at ICU admission have consistently been associated with the development of thrombocytopenia [8–12]. Other baseline conditions which have been associated with an increased risk of thrombocytopenia include onco-haematological neoplasms and liver disease. The most common acute condition in patients developing thrombocytopenia in the ICU is sepsis [8, 11–13]. In the PLOT-ICU study, septic shock increased the risk of severe thrombocytopenia with an odds ratio of 6.90 (2.82–16.89) in adjusted analyses [8].

## Diagnosis of thrombocytopenia

The mechanisms responsible for thrombocytopenia in critically ill patients largely depend on the underlying conditions and the current clinical setting (Table 1). ICU-acquired thrombocytopenia is mainly ascribed to various peripheral mechanisms, including dilution, splenic sequestration, destruction, and consumption. Bone marrow failure, due to both malignant and non-malignant disorders and recent cytotoxic chemotherapy, implies hypoproliferative thrombocytopenia. Furthermore, relative deficiencies in haematologic growth factors such as thrombopoietin, the primary regulator of platelet production, may limit the thrombopoietic response in critically ill patients [14].

**Table 1:** Diagnostic work-up of thrombocytopenia in the critically ill patient

Clinical conditions	Alleged mechanisms of thrombocytopenia	Main relevant investigations
*Medical background*		
Onco-hematological neoplasms Cytotoxic chemotherapy	Bone marrow suppression	Most often obvious from the medical history Bone marrow explorations
Liver cirrhosis	Hypersplenism Hypoproliferative (alcohol toxicity, vitamin deficiencies)	Dosage of vitamins B9 and B12
Pregnancy and peri-partum	Thrombotic microangiopathy HELLP syndrome TTP HUS	Liver blood tests Haemolysis markers with schistocytes Proteinuria Ratio sFlt-1/PlGF^a^ ADAMTS13 activity Exploration of complement inhibition pathways
Post-partum haemorrhage	Dilution, consumption Disseminated intravascular coagulation	Haemostasis tests with quantification of D-dimers
Hemorrhagic shock, hemorrhagic surgery	Dilution, consumption	Haemostasis tests
Travel from endemic areas	Malaria Dengue fever	Thin and thick blood smears, malaria antigen Serology, PCR, ELISA test
Sepsis	Disseminated intravascular coagulation Haemophagocytosis Immune-mediated destruction	Haemostasis tests with quantification of D-dimers
Acute coronary syndrome	Anti GPIIb/IIIa treatment	
Extracorporeal circulations	Membrane clotting	
Sickle cell disease	Bone marrow necrosis	Bone marrow explorations
*Associated organ dysfunctions*		
Neurological manifestations	Thrombotic microangiopathy TTP, malignant hypertension	Cerebral CT-scan^b^ ADAMTS13 activity
Acute kidney injury	Thrombotic microangiopathy HUS, malignant hypertension, TTP, scleroderma renal crisis	ADAMTS13 activity Exploration of complement inhibition pathways
Venous and/or arterial thrombosis	Heparin-induced thrombocytopenia	Anti-PF4 antibodies^c^
	Catastrophic anti-phospholid syndrome	APTT coagulation test Anti-phospholipid and anti-β2 GPI antibodies
	Intravascular disseminated coagulation	Haemostasis tests with quantification of D-dimers
*Biological features*		
Severe isolated thrombocytopenia	Spurious thrombocytopenia	Blood smears Platelet count on citrated samples
	Auto-immune or drug-induced thrombocytopenia	Viral serologies HIV test
Associated anemia and/or leuconeutropenia Pancytopenia Abnormal circulating leukocytes	Bone marrow malignant infiltration Haemophagocytic lymphohistiocytosis Aplastic anemia	Bone marrow explorations
Major macrocytosis Hypersegmented neutrophils	Vitamin B9/B12 deficiencies	Dosage of vitamins B9 and B12
Mononucleosis syndrome	Viral infections, toxoplasmosis, HIV infection	Serologies HIV test
Haemolysis	Thrombotic microangiopathy TTP, HUS, malignant hypertension, renal scleroderma crisis Evans syndrome	ADAMTS13 activity Exploration of complement inhibition pathways Direct antiglobulin test
Bone marrow haemophagocytosis	Haemophagocytic lymphohistiocytosis	Biological components of the H-score

*ADAMTS13* a disintegrin and metalloprotease with thrombospondin type 1 repeats, member 13, *APTT* activated partial thromboplastin time, *HELLP* haemolysis, liver enzymes, low platelets, *HUS* haemolytic uremic syndrome, *PF4* platelet factor 4, *PlGF* placental growth factor, *sFlt-1* fms-like tyrosine kinase 1, *TTP* thrombotic thrombocytopenic purpura

^a^A low sFlt-1/PlGF ratio (typically < 38) allows ruling out pre-eclampsia

^b^To rule out intracranial haemorrhage

^c^Performs with high negative predictive value. Positivity of anti-PF4 antibodies imposes further confirmatory platelet aggregation tests

### Spurious thrombocytopenia

Some technical artefacts may impair the measurement of platelet counts owing to ex vivo platelet aggregation. When detected in an automated haemocytometer, severe thrombocytopenia must be controlled with blood smears to identify platelet aggregates [15]. Spurious thrombocytopenia is often ascribed to insufficient anticoagulation within sampling tubes. An alternative phenomenon of pseudo-thrombocytopenia is related to ethylenediamine tetraacetic acid (EDTA)-induced aggregation of platelets as a consequence of antiplatelet antibodies, which recognise platelet antigens on the platelet membrane when modified by EDTA [16]. Platelet count enumeration in citrated samples usually resolves the clumping, but platelet counts may be significantly underestimated by 10–20% compared to EDTA samples [17].

### Diagnostic workup

Thrombocytopenia in critically ill patients is usually multifactorial. The medical background, including comorbidities and ongoing acute conditions, most often allows an accurate assessment of the causes and mechanisms of thrombocytopenia without further extensive investigations (Table 1). In patients with haemorrhagic surgery or trauma, thrombocytopenia relies on consumption coagulopathy and dilution resulting from aggressive fluid resuscitation.

In non-surgical patients, thrombocytopenia present at the time of ICU admission can result from underlying comorbid conditions, including haematological malignancies, cytotoxic chemotherapy, auto-immune diseases, chronic alcoholism and vitamin deficiencies. As for the latter, when assessed in a systematic diagnostic work-up, the prevalence of folate deficiency was 33% in ICU patients with absolute or relative thrombocytopenia, whereas B12 deficiency was very uncommon [18]. ICU-acquired or ICU-aggravated thrombocytopenia can generally be ascribed to the ongoing acute process (sepsis, haemorrhage) and/or treatments and procedures (drug-induced thrombocytopenia, renal replacement therapy, extracorporeal membrane oxygenation). Also, thrombocytopenia may indicate the presence of disseminated intravascular coagulation (DIC), manifested by systemic activation of coagulation with consumption of coagulation factors and platelets and generation of fibrin, along with variable fibrinolytic activity. The biological and clinical presentations of DIC are rather dependent on the underlying disease. Sepsis-associated DIC exhibits a thrombotic phenotype where fibrinolysis is typically suppressed and likely contributes to organ dysfunctions. DIC associated with haematological malignancies exhibits a fibrinolytic phenotype and exposes patients to bleeding complications [19].

### Indications for bone marrow explorations

Bone marrow aspirates, obtained by bone puncture of sternum or iliac crest, provide unique quantitative and qualitative analyses of medullary precursors. A rich bone marrow with an abundance of megakaryocytes points towards peripheral mechanisms. Conversely, the absence of megakaryocyte lineage, dystrophic megakaryocytes, and malignant infiltration suggest hypoproliferative mechanisms. Haemophagocytosis is a common cytological pattern in the bone marrow of critically ill patients and is often associated with sepsis and transfusions [20]. Features of haemophagocytosis raise the question of the diagnosis of haemophagocytic lymphohistiocytosis, which relies not only on cytopenia but also on alternative clinical and biological manifestations [21].

The diagnostic yield of bone marrow explorations in the ICU has been assessed through a prospective diagnostic study of critically ill patients with thrombocytopenia (here defined as platelet count < 100 × 10^9^/L or relative decrease by 30%). Bone marrow aspirates were performed in 208 patients. Megakaryocytes were present in 93% of bone marrow smears. However, the diagnostic and therapeutic yields of such a systematic approach were relatively poor and resulted in new information in 22% of cases, with further implications in management in 9% only [18]. Bone marrow explorations should, therefore, be restricted to absolute thrombocytopenia associated with non-regenerative anaemia and/or leukoneutropenia and/or the presence of abnormal circulating cells and, more generally, when the mechanisms of thrombocytopenia remain very unclear. In case of inadequate or failed aspiration, a bone marrow trephine biopsy taken from the hip bone may occasionally be required for the definite diagnosis of aplastic anaemia and myelofibrosis and for the staging of lymphoma.

### Sepsis

Sepsis is a prominent determinant of ICU-acquired thrombocytopenia mostly ascribed to endothelial damage and sepsis-associated coagulopathy [22]. Alternative mechanisms have been suggested, including impaired thrombopoiesis owing to dampened production of thrombopoietin, haemophagocytosis and immune-mediated destruction of platelets [14, 23, 24]. In addition, sepsis has been shown to cause platelet dysfunction, including reduced aggregation capacities [25].

### Thrombotic microangiopathy

Thrombotic microangiopathies encompass various diseases where thrombocytopenia results from extensive thrombotic mechanisms within the microcirculation. Thrombotic microangiopathies include the classical thrombotic thrombocytopenic purpura (TTP) and haemolytic uremic syndrome (HUS), as well as alternative disorders such as malignant hypertension, catastrophic antiphospholipid syndrome, scleroderma renal crisis and cancer- and transplantation-associated thrombotic microangiopathy. Although the syndrome refers to histological patterns, the diagnosis of thrombotic microangiopathy primarily relies on haematological features such as the association of peripheral thrombocytopenia and schistocytic haemolytic anaemia. Associated organ failures may overlap but typically include neurological and cardiac manifestations in TTP and renal dysfunction in HUS. TTP and HUS are caused by different pathophysiological mechanisms: through inherited or acquired deficiency in the vWF-cleaving protease ADAMTS13 (a disintegrin and metalloprotease with thrombospondin type 1 repeats, member 13) in TTP, endothelial injury related to Shigatoxin-producing *Enterobacteriaceae* in typical post-diarrhoea HUS and unleashed complement activation in atypical HUS. Owing to the hazard of fast deterioration and sudden death, TTP represents a major diagnostic and therapeutic challenge for intensivists [26]. The peripartum period encompasses various conditions, often associated, including post-partum haemorrhage, disseminated intravascular coagulation and HELLP (Haemolysis, Elevated Liver enzymes and Low Platelet count) syndrome as thrombotic microangiopathy complicating pre-eclampsia. However, the endothelial injury inherent to pregnancy and peri-partum may also precipitate TTP and HUS [27].

## Prognostic value of thrombocytopenia

Most studies consistently show that thrombocytopenia is associated with worse outcomes in a dose–response manner. Although it has been established that ICU patients with thrombocytopenia have higher rates of bleeding, the association between thrombocytopenia and higher mortality or fewer days alive without the use of life-support is also found in non-bleeding patients [7, 28, 29]. This is especially true in patients with severe thrombocytopenia (<50 × 10^9^/L), who have significantly higher mortality and are more likely to experience severe or debilitating bleeding complications compared with patients with no or less severe thrombocytopenia [8]. However, the association with mortality does not necessarily represent a causal relationship, and thrombocytopenia should most often be considered as a marker of severity (e.g. bone marrow failure due to malignancy) rather than the direct cause of the increased mortality. Although the increased risk of bleeding seen in patients with severe thrombocytopenia may contribute to mortality, this is also likely to be influenced by underlying conditions, such as haematological malignancy or trauma [30, 31].

## Bleeding risk assessment

Different scales for bleeding severity assessment have been developed depending on clinical settings. The most used assessment tool for grading bleeding severity is derived from the World Health Organization (WHO) scale, which ranges from 1 to 4 [(1) minor bleeding; (2) mild bleeding; (3) severe bleeding; (4) debilitating bleeding] [8]. A sound relationship between platelet count and bleeding has long been established in non-critically ill onco-haematological patients with hypoproliferative thrombocytopenia [32, 33]. A dramatic increase in the incidence of severe to debilitating bleeding is indeed observed when platelet count drops below 10 × 10^9^/L. Notably, the risk of bleeding in this setting is also driven by additional mechanisms, owing to altered haemostatic functions of platelets as well as damage to endothelial cells or associated coagulopathy [33, 34].

Most studies have reported higher rates of bleeding in critically ill patients with thrombocytopenia. ﻿In the PLOT-ICU cohort study, 27.6% of thrombocytopenic patients bled in the ICU, of whom 57.6% had severe or debilitating bleeding (WHO grade 3 or 4) [8]. In contrast, only 7.3% of patients without thrombocytopenia bled in the ICU, most of whom (81.2%) had minor or mild bleeding (WHO grade 1 or 2). In a French cohort of patients with septic shock, the incidence of ICU-acquired severe-to-debilitating bleeding was 14.4%, 15.3% and 20.3% in patients with mild, moderate and severe thrombocytopenia, respectively, vs. 3.6% in non-thrombocytopenic patients [12].

**Fig. 1:**
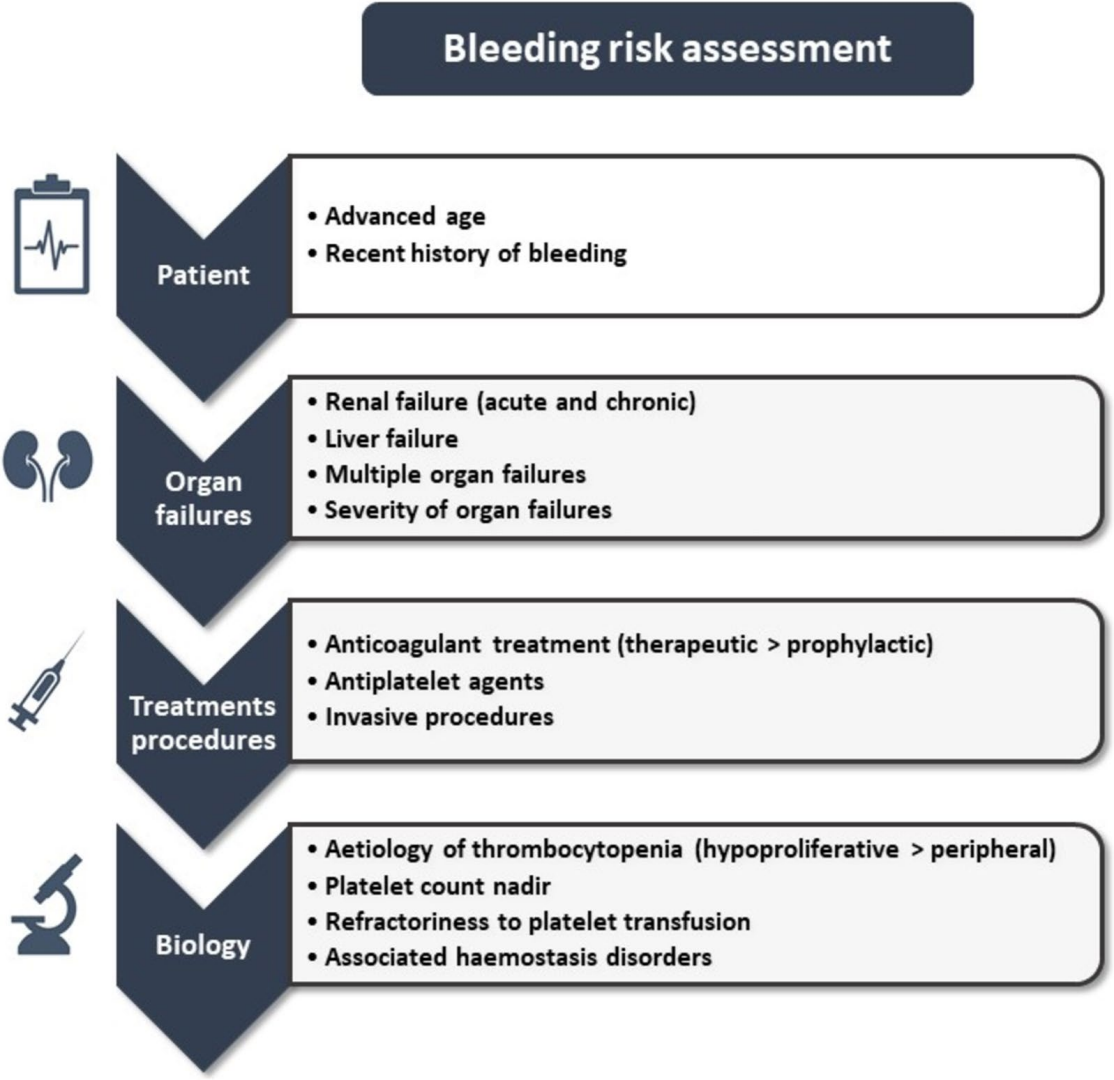
Risk factors of bleeding in thrombocytopenic critically ill patients

It is noteworthy that thrombocytopenia is not consistently retained in multivariate prediction models for ICU-acquired bleeding [12, 30, 35, 36] and that the common correlation of platelet count with severe haemorrhage, as observed in non-critically ill patients with onco-haematological malignancies, appears less consistent in critically ill patients [8, 18, 35]. These data suggest that the risk of bleeding is not solely dependent on platelet count but may depend on both the mechanism of thrombocytopenia and alternative mechanisms involved in critical illness. Hypoproliferative thrombocytopenia generally harbours a higher risk of bleeding than peripheral thrombocytopenia, which is characterised by sustained platelet production and preserved haemostatic functions. In critically ill patients with haematological malignancies, hence with a high frequency of hypoproliferative thrombocytopenia, the risk of bleeding was approximately three times higher compared to their non-critically ill counterparts [37]. Accordingly, the overall and major bleeding rates among 116 critically ill patients with acute leukaemia or myelodysplastic syndromes were considerably high, 57% and 33%, respectively [30]. Several additional factors concur with the increased risk of bleeding in ICU settings, including alternative coagulation disorders, anticoagulant and antiplatelet agents, co-existing liver disease, and the extent of organ failures (Fig. 1). Interestingly, kidney dysfunction, as assessed by chronic or acute renal failure or by the requirement of renal replacement therapy, has been consistently linked to ICU-acquired bleeding, possibly owing to defective platelet aggregation related to hyperuremia [35, 36, 38]. Although DIC is primarily ascribed to underlying hypercoagulable processes, oozing at wounds, around central lines, and catheters are more common clinical manifestations associated with platelet deficiencies and coagulation factors [39]. A recent bleeding episode should be considered a warning event associated with further recurrence or new-onset bleeding events [36].

Besides standard laboratory haemostasis tests, viscoelastic tests such as thromboelastography (TEG) and rotational thromboelastometry (ROTEM), available as point-of-care assays provide a comprehensive view of clot formation and lysis with regard to early platelet aggregation, but also to the coagulation/anticoagulation and fibrinogenesis/fibrinolysis balances. The overall stability of the clot is represented by the ‘maximum amplitude’ value in TEG, corresponding to the ‘maximum clot firmness’ in ROTEM, both dependent on platelets and fibrin. Although the use of viscoelastic tests has been found to save time in the management of trauma-associated bleeding [40], their application to other conditions remains controversial. For instance, most patients with sepsis typically present with a maximum amplitude within or above the reference range despite many being thrombocytopenic [41]. The ability of TEG to identify patients at high risk of bleeding is very low, both in patients with sepsis and in critically ill patients with haematological malignancies [42].

## Platelet transfusions

Platelet transfusion is given to prevent or treat bleeding in four main settings: (1) Prevention of bleeding in patients with severe thrombocytopenia, (2) Prior to invasive procedures in thrombocytopenic patients, (3) Treatment of WHO grade 2–4 bleeding in thrombocytopenic patients, and (4) Management of massive bleeding with haemorrhagic shock regardless of platelet count (Table 2) [43].

**Table 2:** Suggested platelet transfusion thresholds in critically ill patients

Indications	Platelet count threshold (x 10^9^/L)	Clinical setting
Prophylactic platelet transfusion	10 20 50	Hypoproliferative and peripheral thrombocytopenia If judged to have additional risk factors for bleeding^a^ Therapeutic-dose anticoagulation treatment
Pre-procedural platelet transfusion	10–20 40–50 80 100	Ultrasound-guided CVC placement (in compressible sites) Lumbar puncture, non-neuroaxial surgery Epidural analgesia Neuroaxial surgery
Therapeutic platelet transfusion	30 50 100	Mild bleeding Severe to debilitating bleeding Intracranial haemorrhage
No platelet transfusion		Thrombotic microangiopathies (TTP+++, HUS, HELLP syndrome, DIC, HIT, CAPS)^b^ Immune thrombocytopenic purpura^b^ Reversal of antiplatelet agents in intracranial haemorrhage

The proposed thresholds should be adapted to the individual patient’s condition and the associated risk factors for bleeding. Adapted from (59–61, 69, 70, 75, 78)

*CAPS* catastrophic antiphospholipid syndrome, *CVC* central venous catheter, *DIC* disseminated intravascular coagulation, *HELLP* haemolysis, liver enzymes, low platelets, *HIT* heparin-induced thrombocytopenia, *HUS* haemolytic uremic syndrome, *TTP* thrombotic thrombocytopenic purpura

^a^Such as fever ≥ 38.5 °C, infection, severe mucositis, lesion at risk of bleeding, fast decrease in platelet count

^b^No indication for prophylactic transfusion. Therapeutic transfusion if severe or debilitating bleeding

### Platelet products

There is high variability in platelet processing methods across countries with differences in the type of additive solution, the pathogen inactivation method, the storage duration (usually between 5 and 7 days), and platelet dose and volume of platelet concentrates. Platelets are stored at room temperature (20–24 °C) under slow and constant agitation. The short shelf-life of platelet concentrates puts them at risk of shortage and waste [44]. Platelet concentrates can be prepared from a single donor by apheresis or be pooled from whole blood from several donors to amount to an adult therapeutic platelet dose [44]. The efficacy of single-donor apheresis platelets and whole blood-derived platelets are similar, and the choice of products mainly depends on their availability unless specific indications are present, such as documented anti-HLA immunisation.

Alternative platelet products include cold-stored, frozen and lyophilised platelets [45]. Cold-stored platelets are stored at 1–6 °C for up to 14 days. Although cold-stored platelet concentrates result in lower post-transfusion platelet increments, they exhibit increased haemostatic capacities. In a recent phase 2 multicentre randomised controlled trial, the early administration of cold-stored platelets in severely injured patients was safe and did not result in significantly different 24-h mortality compared to standard care [46]. Frozen platelets could increase availability, especially in remote areas, as they can be stored for several years. Lyophilised platelets are under development. Here, platelets are treated either with paraformaldehyde or trehalose to stabilise the platelet membrane, allowing storage at room temperature for several years before being reconstituted with sterile water [45]. Such alternative platelet products are only considered for therapeutic transfusion and are currently only available in very few settings, such as the treatment of armed services personnel.

### Side effects of platelet transfusions

In a post-hoc analysis of a large randomised trial on platelet dosing strategies in haematology patients, up to 10% of platelet transfusions were associated with adverse events, that were mainly fever for 66% of them [47]. Although room temperature preserves some platelet function and maximises platelet recirculation, it also facilitates bacterial growth and causes morphological and biochemical changes to the platelets, so-called “storage lesions”. Improvements in platelet manufacturing include systematic pathogen reduction and plasma replacement by platelet additive solution. In France, the pathogen inactivation method includes the synthetic psoralene compound amotosalen in combination with ultraviolet A (UVA) light treatment since November 2017, which resulted in a steep reduction in transfusion-transmitted blood infections [48]. Importantly, as systematic pathogen reduction technology does not prevent the transmission of non-enveloped viruses, the risk of hepatitis E and A virus transmission persists [48]. Plasma replacement contributed to the drop in transfusion-related acute lung injury secondary to platelet transfusion in France [49]. Other complications include circulatory overload, allergic reaction, anti-human leukocyte antigen (HLA) and anti-human platelet antigen (HPA) allo-immunisation. Platelet concentrates may also harbour immunomodulatory properties, as suggested by the consistent association of platelet transfusions with the further development of hospital-acquired infections [50–52]. The safety of prophylactic platelet transfusions was challenged in the randomised trial PLANET2, which assessed two platelet count thresholds (25 × 10^9^/L vs. 50 × 10^9^/L) in pre-term neonates [53]. Unexpectedly, a higher risk of bleeding and a trend towards increased mortality was observed in the higher threshold arm. Though this may not apply to adult critically ill patients, such paradoxical findings in neonates suggest that bioreactive components from platelet concentrates may have promoted inflammation and tissue injury.

### Indications for prophylactic platelet transfusion

In the absence of a completed randomised controlled trial comparing different platelet count thresholds for prophylactic platelet transfusion in the ICU, the transfusion strategy in critically ill patients remains largely derived from the studies performed in stable haematology patients. Two randomised controlled trials assessed prophylactic (triggered by a platelet count below 10 × 10^9^/L) vs. therapeutic-only platelet transfusion strategies in patients with haematological malignancies who received intensive chemotherapy for acute leukaemia or autologous hematopoietic stem cell transplantation. A decrease in grade 2–4 bleeding was observed in patients receiving prophylactic platelet transfusion as compared to the therapeutic-only arm [54, 55]. Most importantly, intracranial haemorrhage was more frequent in the subgroup of patients with acute leukaemia allocated to the therapeutic-only strategy. This suggests that not only the nadir platelet count but also the duration of thrombocytopenia is a determinant of severe bleeding. Platelet count transfusion thresholds of 10 × 10^9^/L vs. 20 × 10^9^/L have been assessed in patients with haematological malignancies, where the restrictive arms demonstrated significant decreases in platelet transfusions without increasing the risk of bleeding [56–59]. Most guidelines currently recommend platelet count transfusion thresholds of 10–20 × 10^9^/L, taking into account the presence of factors known to shorten the platelet lifespan or to increase the risk of bleeding, including fever > 38.5 °C, ongoing infection, high blood pressure, severe mucositis, and a sharp drop in platelet count within the last 72 h [60].

Critically ill patients have different risk factors for thrombocytopenia, and they also have additional risk factors for bleeding, likely hampering the generalisation of platelet transfusion practices in haematology patients to ICU patients. The clinical practice guidelines from the European Society of Intensive Care Medicine (ESICM) suggest not using prophylactic platelet transfusion unless the platelet count falls below 10 × 10^9^/L in non-bleeding critically ill adults, though based on very low certainty of evidence [61]. Prophylactic platelet transfusion is usually contra-indicated in patients with thrombotic microangiopathies, especially in TTP, where platelet transfusions have occasionally been associated with thrombotic events and dramatic clinical deterioration [62, 63]. This restriction may extend to alternative thrombocytopenic thrombotic disorders [63].

### Post-transfusion platelet response

The yield of platelet transfusion is typically assessed at the bedside by the absolute increment in platelet count. However, more accurate measurements exist. The corrected count increment (CCI = (post-transfusion platelet count − pre-transfusion platelet count) x body surface/number of platelets within concentrate) and the platelet transfusion recovery (PTR = (post-transfusion platelet count − pre-transfusion platelet count) x bodyweight × 0.075/number of platelets within concentrate) adjust for both the administered platelet dose and the estimated blood volume [64]. A poor platelet response, as assessed by a CCI < 7 on the day after transfusion, has been observed in 50–75% of transfusion episodes in critically ill patients [65, 66]. Factors independently associated with poor platelet increment in critically ill patients included higher patient severity scores, sepsis at admission, underlying haematological malignancy, storage duration of platelet concentrates, fever and antibiotic therapy at the time of the transfusion [65, 66]. Refractoriness is characterised by a poor transfusion yield (either CCI < 7 or PTR less than 20%, obtained within 1 h of transfusion completion) following two consecutive transfusion episodes with ABO-compatible platelet concentrates featuring short storage time (less than 3 days). Refractoriness to platelet transfusion, more broadly assessed by the closest post-transfusion platelet count, has been found to occur in 23.3–54.8% of critically ill patients and has been associated with increased risk of bleeding [65, 67]. Both immune and non-immune factors may result in transfusion refractoriness. Immune mechanisms are documented in 20% of refractoriness in patients with iterative platelet transfusions and include allo-immunisation to human leukocyte antigen (HLA) or human platelet antigen (HPA), ABO incompatibility, anti-platelet autoantibodies and drug-related antibodies. Non-immune reasons rely on shortened platelet lifespan, including spleen enlargement, pregnancy, disseminated intravascular coagulation, graft-versus-host disease, and sinusoidal obstruction syndrome [64, 65, 67, 68]. The transfusion strategy in patients with immune-mediated refractoriness relies on HLA- or HPA-matched, or crossmatch-compatible platelet concentrates [68]. A therapeutic-only transfusion strategy, i.e. to administer platelet transfusion only in case of overt significant bleeding of grade 2 or higher, may also be considered in this setting.

### Indications for pre-procedural platelet transfusion

The platelet count transfusion threshold to secure invasive procedures remains based on a low level of evidence. The ESICM guidelines recommend no transfusion before invasive procedure when the platelet count is above 100 × 10^9^/L and before central venous catheter (CVC) insertion and percutaneous tracheotomy when the platelet count is between 50 × 10^9^/L and 100 × 10^9^/L [61]. The French guidelines advocate a minimal platelet count of 50 × 10^9^/L for lumbar puncture, liver biopsy, bronchoscopy, osteomedullary biopsy, and a platelet count increased to at least 80 × 10^9^/L for epidural analgesia insertion or ablation [60]. However, the supporting evidence was of low certainty and based on observational studies and small randomised trials at high risk of biases. A recent trial randomised thrombocytopenic patients with platelet counts between 10 and 50 × 10^9^/L, hospitalised either in the ICU or in the haematology ward, to receive one platelet transfusion vs. none prior to ultrasound-guided placing of a CVC [69]. Patients randomised to the no-transfusion group had more catheter-related bleeding than those who received a prophylactic platelet transfusion (OR 2.45, 90% CI 1.27–4.70). However, this difference was not found in the subgroup of only ICU patients. Therefore, these results suggest that ultrasound-guided insertion of CVC in compressible sites can be performed safely in the ICU without pre-procedural platelet transfusion.

### Indications for therapeutic platelet transfusion

High-quality evidence to guide clinical practice regarding therapeutic platelet transfusion for active bleeding is lacking. The British Society of Haematology specifies a platelet count threshold of 30 × 10^9^/L in case of minor bleeding [70]. The guidelines for the management of major bleeding and coagulopathy following trauma recommend maintaining a platelet count above 50 × 10^9^/L and above 100 × 10^9^/L in case of major bleeding and/or brain injury [71]. In case of massive bleeding in trauma, early administration of platelet transfusion might be beneficial. In the PROPPR trial that compared 1:1:1 vs. 1:1:2 ratios of platelet: plasma: red blood cells, death due to exsanguination was less frequent in patients randomised in the 1:1:1 ratio group (i.e. receiving higher amounts of platelets and plasma), yet without difference in 24-h mortality [72]. Accordingly, a secondary analysis of this trial found that early platelet transfusion was associated with reduced 24-h and 30-day mortality rates (5.8% vs. 16.9%; *p* = 0.05, and 9.5% vs. 20.2%; *p* = 0.05, respectively) [73]. In this study, early platelet transfusion also led to better achievement of haemostasis (94.9% vs. 73.4%; *p* = 0.01) and was less often associated with death due to exsanguination (1.5% vs. 12.9%; *p* < 0.01) [73]. Hamada et al. reported in a retrospective multicentre observational study that included 19,596 trauma patients a significant decrease in 24-h all-cause mortality in patients receiving early (<6 h) platelet transfusion (OR 0.52, 95% CI 0.34–0.79; *p* < 0.05) as compared to patients with no or late platelet transfusion [31]. The management of non-trauma critically ill patients with severe bleeding is derived mainly from the transfusion strategy set in trauma patients.

In addition, platelet transfusions have been previously considered to offset the effect of antiplatelet agents in treating severe bleeding. In a trial where adults suffering from intracranial haemorrhage with a Glasgow Coma Scale score between 8 and 15 and under antiplatelet agents were randomised to receive one platelet transfusion or none, transfused patients unexpectedly exhibited worse outcomes of death or dependence at 3 months [74], strongly arguing against this strategy [75].

## Thrombopoiesis-stimulating agents

Regardless of underlying aetiologies, thrombocytopenia is often associated with inadequate thrombopoietic response. Folic acid supplementation is indicated for treating absolute folate deficiency and can be considered to prevent any relative deficiency resulting from enhanced hematopoietic response towards sustained haemolysis and platelet consumption. Thrombopoiesis-stimulating agents stimulate megakaryocyte differentiation and proliferation to promote platelet production. Thrombopoiesis-stimulating agents have emerged as promising treatments for various causes of thrombocytopenia, including myelodysplastic syndromes, cytotoxic chemotherapy, immune thrombocytopenia and cirrhosis. Experience in critically ill patients is limited, although a meta-analysis provided encouraging results in sepsis patients where recombinant human thrombopoietin increased platelet count after 7 days and decreased the need for platelet transfusion [76]. Nonetheless, the potential for thrombosis is of concern, and the use of thrombopoiesis-stimulating agents in critically ill patients remains controversial, although it has been mentioned as a possibility in recent Chinese guidelines [77].

## Conclusion

Thrombocytopenia in critically ill patients is very frequent, either already present at admission or acquired or worsened during the ICU stay. The implications for intensivists are manifold in terms of diagnosis, prognostic value, assessment of the risk of bleeding and indications of platelet transfusions. The impact of thrombocytopenia on bleeding remains uncertain in critically ill patients who exhibit multiple risk factors, including vascular and epithelial damage, coagulation dysfunction and exposure to numerous invasive procedures. Platelet transfusion guidelines in ICU are often based on low quality of evidence, especially for prophylactic transfusion. Platelets remain valuable and perishable resources with potential adverse events, making it crucial to better define their indications to avoid unnecessary transfusions and improve patients’ outcomes.

## Data Availability

Not applicable.
